# Beneficial effects of simultaneously targeting calorie intake and calorie efficiency in diet-induced obese mice

**DOI:** 10.1042/CS20231016

**Published:** 2024-02-19

**Authors:** Sing-Young Chen, Aiden J. Telfser, Ellen M. Olzomer, Calum S. Vancuylenberg, Mingyan Zhou, Martina Beretta, Catherine Li, Stephanie J. Alexopoulos, Nigel Turner, Frances L. Byrne, Webster L. Santos, Kyle L. Hoehn

**Affiliations:** 1 School of Biotechnology and Biomolecular Sciences, University of New South Wales, Sydney, NSW 2052, Australia; 2 Cellular Bioenergetics Laboratory, Victor Chang Cardiac Research Institute, Darlinghurst, NSW 2010, Australia; 3 School of Biomedical Sciences, University of New South Wales, Sydney, NSW 2052, Australia; 4 Department of Chemistry and Virginia Tech Centre for Drug Discovery, Virginia Tech, Blacksburg, VA 24061, U.S.A.

**Keywords:** drug effects, glucose homeostasis, mouse models

## Abstract

Semaglutide is an anti-diabetes and weight loss drug that decreases food intake, slows gastric emptying, and increases insulin secretion. Patients begin treatment with low-dose semaglutide and increase dosage over time as efficacy plateaus. With increasing dosage, there is also greater incidence of gastrointestinal side effects. One reason for the plateau in semaglutide efficacy despite continued low food intake is due to compensatory actions whereby the body becomes more metabolically efficient to defend against further weight loss. Mitochondrial uncoupler drugs decrease metabolic efficiency, therefore we sought to investigate the combination therapy of semaglutide with the mitochondrial uncoupler BAM15 in diet-induced obese mice. Mice were fed high-fat western diet (WD) and stratified into six treatment groups including WD control, BAM15, low-dose semaglutide without or with BAM15, and high-dose semaglutide without or with BAM15. Combining BAM15 with either semaglutide dose decreased body fat and liver triglycerides, which was not achieved by any monotherapy, while high-dose semaglutide with BAM15 had the greatest effect on glucose homeostasis. This study demonstrates a novel approach to improve weight loss without loss of lean mass and improve glucose control by simultaneously targeting energy intake and energy efficiency. Such a combination may decrease the need for semaglutide dose escalation and hence minimize potential gastrointestinal side effects.

## Introduction

Obesity is a major health concern because it strongly predisposes individuals to diseases such as Type 2 diabetes, non-alcoholic fatty liver disease, cardiovascular disease, and cancer [1,2]. The ideal intervention for obesity is lifestyle modification to decrease calorie intake while retaining diet quality and increasing exercise frequency and intensity in a sustainable manner. However, lifestyle intervention alone has been vastly inadequate for successfully treating obesity on a global scale. Long-term calorie restriction is difficult to maintain; for example, in the CALERIE trial, patients stratified to the 25% calorie restriction group were averaging only 9.1% calorie restriction after 6 months [3]. Calorie restriction also involves the unwanted side effects of decreased resting energy expenditure (REE) [3] and loss of lean muscle mass and bone density [4]. Exercise intervention confers many physiological benefits, but its weight loss efficacy is hampered by the difficulty of generating a sufficient calorie deficit, compensatory increases in appetite and food intake, and decreased expenditure through non-exercise activities [5–7]. Exercise intensity may also be limited by patient mobility and other health-related constraints. A more invasive treatment for obesity is bariatric surgery, which has shown greater success in weight control, but is costly, invasive, and inaccessible to most patients [8–12]. Therefore, adjunct pharmacotherapy may be required to manage the global obesity crisis.

In recent years, glucagon-like peptide 1 receptor agonists (GLP-1-RAs) have become commonly used pharmacotherapies for obesity and type 2 diabetes. In particular, semaglutide, a long-acting GLP-1-RA, has recently been approved for both glucose-lowering and weight loss [13,14]. Semaglutide mimics native GLP-1 to increase satiety and decrease food intake, while also stimulating insulin secretion and improving insulin sensitivity [15,16], thus promoting a calorie deficit and improving blood glucose homeostasis. Semaglutide was first approved as a glucose-lowering drug for people with type 2 diabetes at doses up to 1 mg by subcutaneous injection once weekly, which was sufficient to decrease mean HbA1c by 1.5–1.6% after 56 weeks of treatment [17,18]. However, to promote effective weight loss, patients who are prescribed semaglutide for obesity are typically titrated up to a dose of 2.4 mg weekly, which has stronger efficacy than semaglutide 1.0 mg weekly [19]. Gradual titration to increase the dosage is important to build tolerability to adverse gastrointestinal effects, as dose escalation frequently results in nausea, vomiting, diarrhoea, and constipation [20]. While these effects are reported to subside over time for many patients, some patients discontinue semaglutide usage as they are unable to tolerate the persistent gastrointestinal side effects [17–19,21–23]. Therefore, it would be of great value to develop a treatment strategy that can achieve effective weight loss using a lower dose of semaglutide.

The weight loss effects of semaglutide are achieved in large part by decreasing energy intake. An alternative strategy for weight loss is to increase energy expenditure by mitochondrial uncoupling [24]. Mitochondrial uncoupling decreases metabolic efficiency so that more nutrients are oxidised to generate a given amount of ATP [25]. Under normal conditions, cells undergo physiological mitochondrial uncoupling [26], but mitochondrial uncoupling can also be induced pharmacologically using small molecule drugs [24]. Hence, combining GLP-1-RAs and mitochondrial uncouplers may be effective by both decreasing energy intake and decreasing the amount of energy derived from food. However, it remains unclear whether this combination would be sufficient to overcome the strong compensatory mechanisms that defend against weight loss. Notably, decreasing calorie intake results in compensatory improvements in metabolic efficiency that are robust and difficult to overcome, even by increasing exercise [27,28]. Moreover, it is possible that the addition of a mitochondrial uncoupler to a semaglutide regime may result in compensatory increased appetite and food intake, which has been observed with conventional exercise therapy [29,30].

While no mitochondrial uncouplers are currently approved for obesity, the mitochondrial uncoupler 2,4-dinitrophenol (DNP) was used in humans as a weight loss molecule in the 1930s [31,32]. However, DNP was soon discontinued due to severe adverse side effects, including cataracts, hyperthermia, and even death [33–35]. DNP has multiple actions apart from mitochondrial uncoupling including plasma membrane depolarisation [36,37]. Recently, other uncouplers, including derivatives of DNP, have been developed with good safety profiles in rodents [38–44]. The most clinically advanced molecule HU-6 has undergone a Phase 2a trial for liver fat reduction in humans and was reported to be well-tolerated [45]. HU-6 is not commercially available, but another well-characterized mitochondrial uncoupler BAM15 decreases body weight and improves glucose tolerance in obese mice [43,46,47], without plasma membrane depolarisation [48] or causing hyperthermia in diet-induced obese mice [43,46]. Moreover, published indirect calorimetry experiments support the expected mechanism of action of BAM15; when admixed in diet, BAM15 increased oxygen consumption independent of locomotor activity in high-fat diet-fed mice [43].

Herein, we sought to determine whether combining semaglutide with BAM15 would result in better effects on body weight and blood glucose control in a mouse model of obesity, compared with either drug given alone. Two doses of semaglutide and one dose of BAM15, all with sub-maximal effects, were tested in diet-induced obese mice. Previously, we defined the submaximal BAM15 dose as 0.05% (w/w) admixed in diet as it was compared with higher doses of 0.1% and 0.15% in the same western diet used herein [43]. Therefore, the 0.05% BAM15 dose used in this study is at least 3-fold lower than the maximally effective dose. Similarly, semaglutide studies in diet-induced mice in the literature commonly involve doses of 40–600 µg/kg every 1–3 days [49–58]. Therefore, we tested semaglutide herein at 5 µg/kg (low dose) and 20 µg/kg (high dose) twice weekly, which are 2- to 8-fold lower than the lowest reported effective dose. Combination treatment with BAM15 and the low-dose semaglutide resulted in decreased body fat and liver triglycerides, which was not achieved by any monotherapy, while high-dose semaglutide with BAM15 not only decreased body fat and liver triglycerides but also improved glucose homeostasis. Importantly, the weight loss effects were primarily fat loss without loss of lean mass. These data identify a novel strategy of treating obesity and obesity-related comorbidities by simultaneously targeting energy intake and energy efficiency, a mechanism that is distinct from all other available pharmacological interventions.

## Methods

### Animal husbandry

All experimental procedures were conducted at UNSW and approved by the UNSW Animal Care and Ethics Committee (project approval code : 20/67A). Six-week-old male C57BL/6J mice were purchased from Australian Bio-Resources (Moss Vale, NSW, Australia). Mice were allowed to acclimatise for 1 week prior to any procedures. All mice were housed in pathogen-free conditions at 22°C with a light/dark cycle of 12 h and given *ad libitum* access to water and high-fat western diet (WD).

### Study design

To establish a model of diet-induced obesity, 38 8-week-old male C57BL/6J mice were first conditioned on WD for 5 weeks in cages of up to 5 mice and provided *ad libitum* access to WD. Body weight was measured weekly, and body composition was assessed using the EchoMRI™ Body Composition Analyzer. After the 5-week conditioning period, all mice were single-housed and a pre-treatment glucose tolerance test (GTT) was conducted. Mice were stratified into treatment groups based on fat mass and glucose tolerance. The treatment groups were: WD, BAM15, low-dose 5 µg/kg semaglutide, low-dose semaglutide + BAM15, high-dose 20 µg/kg semaglutide, and high-dose semaglutide + BAM15. There were 8 mice in the WD group and 6 mice in all others. Mice were provided either WD or WD containing BAM15 at 0.05% (w/w), according to their treatment groups. The 0.05% dose has been previously published to achieve mild and submaximal effects on weight loss and blood glucose, in contrast to the higher 0.1% and 0.15% doses that had stronger effects [43]. Twice a week, mice were given a subcutaneous injection of PBS (vehicle) or semaglutide at 5 µg/kg or 20 µg/kg body weight, according to their treatment groups. Food intake was measured every weekday and body composition was measured weekly by EchoMRI. After 4 weeks of treatment, a post-treatment glucose tolerance test was performed. At termination, blood was collected by cardiac puncture under isoflurane anaesthesia and mice were euthanised by cervical dislocation. Serum was extracted by centrifugation at 2000 × ***g*** for 10 min at 4°C and stored at −80°C. Dissected tissues were collected and snap-frozen in liquid nitrogen before storage at -80°C for subsequent analysis.

### Preparation of high-fat western diet

The high-fat western diet was prepared as previously described [43] with ∼45% of calories from fat (lard), 35% from carbohydrates, and 20% from protein. For diet containing BAM15, BAM15 was obtained from Santos Lab, Virginia Tech, U.S.A., and added to WD at 0.05% (w/w). This concentration of BAM15 amounted to approximately 40 mg BAM15 consumed per kg body weight per day.

### Semaglutide administration

Semaglutide was administered by subcutaneous injections twice weekly, at the same time of day between 9 and 10 AM. To prepare the working solution, a 1.34 mg/ml Ozempic (semaglutide) stock solution (Novo Nordisk) was diluted in sterile-filtered phosphate-buffered saline (PBS) to a final concentration of 1.67 or 6.7 µg/ml, to achieve doses of 5 and 20 µg/kg body weight, respectively, when given at 3 µl/kg body weight. Mice that did not receive semaglutide were given a vehicle injection of PBS of equivalent volume.

### Glucose tolerance test

Glucose tolerance tests (GTTs) were performed following a 6-h daytime fast from 8:30 AM to 2:30 PM, 2 days following the most recent injection of semaglutide or vehicle. Mice were administered 33.3% (w/v) glucose solution dissolved in 0.9% sodium chloride and delivered by intraperitoneal injection at 2 g/kg lean mass. Blood glucose levels were measured at the indicated time points using an Accu-chek Performa II glucometer (Roche, Australia). Total area under the curve (AUC) was calculated using the trapezoidal method. Incremental area under the curve was calculated as the area under the curve above baseline, excluding any region of the curve that dropped below baseline for each individual animal.

### Plasma insulin measurements

Fasting blood samples were collected from the tail tip after a 6-h daytime fast using heparinised capillary tubes (Sarstedt, Australia). Plasma was extracted by centrifugation at 2000 × ***g*** for 10 min at 4°C. Plasma insulin concentrations were determined using the Crystal Chem Ultra-Sensitive Mouse Insulin ELISA Kit (Crystal Chem Inc., 90080, U.S.A.), according to the manufacturer’s instructions, except that test samples and standards were incubated overnight.

### Liver triglyceride assay

Triglyceride contents in frozen liver tissue were assessed as previously described [47] using a modified Folch’s method [59].

### Indirect calorimetry

Separate cohorts of 19 9-week-old male C57BL/6J mice were conditioned on WD for 2 weeks, then placed in an Oxymax CLAMS (Columbus Instruments’ Comprehensive Lab Animal Monitoring System, U.S.A.) indirect calorimeter in the Biological Resources Centre at UNSW. After one day of acclimatisation, baseline measurements were taken to measure oxygen consumption and carbon dioxide production, and laser beam breaks were recorded to measure locomotor activity. Air flow rate was recorded at 0.499 L/min over the course of the experiment. Once 24 h of baseline measurements had been acquired, a subset of animals was switched from WD to WD containing 0.05% BAM15, and measurements were taken for a further 24 h. Then, all animals received a subcutaneous dose of vehicle or 20 µg/kg body weight semaglutide before measurements were collected for another 24 h. There were six mice in each group given WD, BAM15 alone, and semaglutide alone, and seven mice in the BAM15 + semaglutide group. Respiratory exchange ratio (RER) was calculated as the ratio of carbon dioxide output over oxygen consumption (*V*CO_2_/*V*O_2_).

### Western blots

Total protein was extracted from powdered frozen liver tissue by homogenisation in lysis buffer (50mM Tris HCl [pH 7.4], 1 mM EDTA, 1 mM EGTA, 10% glycerol, 1% Triton X-100, 50 mM sodium fluoride, 5 mM sodium pyrophosphate, cocktail of protease and phosphatase inhibitors). Homogenate was probe-sonicated on ice, put through a 25 G needle ∼5 times to shear DNA, then centrifuged at 16,000 × ***g*** and 4°C for 10 min, and the supernatants collected. Protein concentrations were quantified using the Pierce Bicinchoninic Acid (BCA) protein assay kit (ThermoScientific, 23225, Australia), according to the manufacturer’s instructions. Protein samples were normalized to 2 μg/μl in lysis buffer and loading buffer. Samples were heated at 65°C, cooled to room temperature, and 30 μg was run on Any kD Mini-Protean TGX Precast gels (Bio-Rad) alongside protein ladder (Seeblue Plus2, ThermoScientific LC5925). Proteins were transferred to nitrocellulose membranes, which were stained with Ponceau S and blocked with 5% (w/v) skim milk. Membranes were incubated at 4°C overnight in antibodies specific for phospho-AMPKα (Thr172) (Cell Signaling Technology, 2535, U.S.A.), acetyl-CoA carboxylase (ACC) (Cell Signaling Technology, 3676, U.S.A.), phospho-ACC (Ser79) (Cell Signaling Technology, 3661, U.S.A.), fatty acid synthase (FAS, Cell Signaling Technology, 3180, U.S.A.), AMPK (Santa Cruz, sc-74461, U.S.A.), and β-actin (A5441, Sigma-Aldrich, Australia). Membranes were then incubated in fluorescent secondary antibodies for 1 h at room temperature, including donkey anti-mouse IgG (AlexaFluor790) (Abcam ab186699) or anti-rabbit IgG (AlexaFluor680) (Abcam ab186692). Imaging was performed on the LI-COR ODYSSEY CLx system (LI-COR, Lincoln, NE, U.S.A.), and densitometry was used to calculate the ratio of phosphorylated protein to total protein. Where phosphorylated and total proteins were run on the different gels (ACC), bands were first normalized to the density of the loading control.

### Fatty acid oxidation enzyme activity assays

To measure 3‐hydroxyacyl‐CoA dehydrogenase (β-HAD) and medium chain acyl-CoA dehydrogenase (MCAD) activity, liver homogenates were prepared by homogenising frozen liver tissue in Tris buffer (50 mM Tris-HCl buffer, 0.1% [v/v] Triton X-100, 1 mM EDTA, pH 7.4) using a pellet pestle homogeniser (Sigma Z359971). Liver homogenates were subjected to three freeze-thaw cycles and then centrifuged at 4600 × ***g*** for 10 min at 4°C and the supernatant collected. To measure β-HAD activity, liver lysates were diluted 1:6 in Tris buffer, then diluted 1:2 in Reaction buffer 1 (50 mM imidazole, 1.2 mM EDTA, 0.3 mM NADH, pH 7.4). In a 96-well plate, 5 µl of the diluted sample was combined with 120 µl Reaction buffer 1 and pre-incubated at 30°C for 4 min. The reaction was initiated with 25 µl of 0.6 mM acetoacetyl-CoA and absorbance readings at 340 nm were taken continuously for 5 min at 30°C. To measure MCAD activity, liver lysates were diluted 1:5 in Tris buffer. In a 96-well plate, 15 µl of sample was added to 110 µl Reaction buffer 2 (100 mM KH_2_PO_4_, 1 mM EDTA, 0.5 mM sodium tetrathionate, 200 µM ferrocenium hexafluorophosphate) and pre-incubated at 30°C for 4 min. The reaction was initiated with 25 µl of 5.6 mM octanyl-CoA and absorbance readings at 300 nm were taken continuously for 5 min at 30°C.

### Statistical analyses

All data points were collected from discrete biological replicates and are presented as the mean ± SD. Significance was determined to be reached when *P*<0.05 using Prism (v9.5.0; GraphPad Software). Selected groups were compared by one-way ANOVA with Sidak’s multiple comparisons test. Selected comparisons included WD compared with each of the other groups, low-dose semaglutide compared with low-dose semaglutide + BAM15, high-dose semaglutide compared with high-dose semaglutide + BAM15, BAM15 compared with low-dose semaglutide + BAM15, and BAM15 compared with high-dose semaglutide + BAM15. To test for differences in food intake, Tukey’s multiple comparisons test was performed to compare each group to every other group. For indirect calorimetry data across three time periods, two-way ANOVA with Sidak’s multiple comparisons was performed, comparing each group to every other group.

## Results

**Figure 1:**
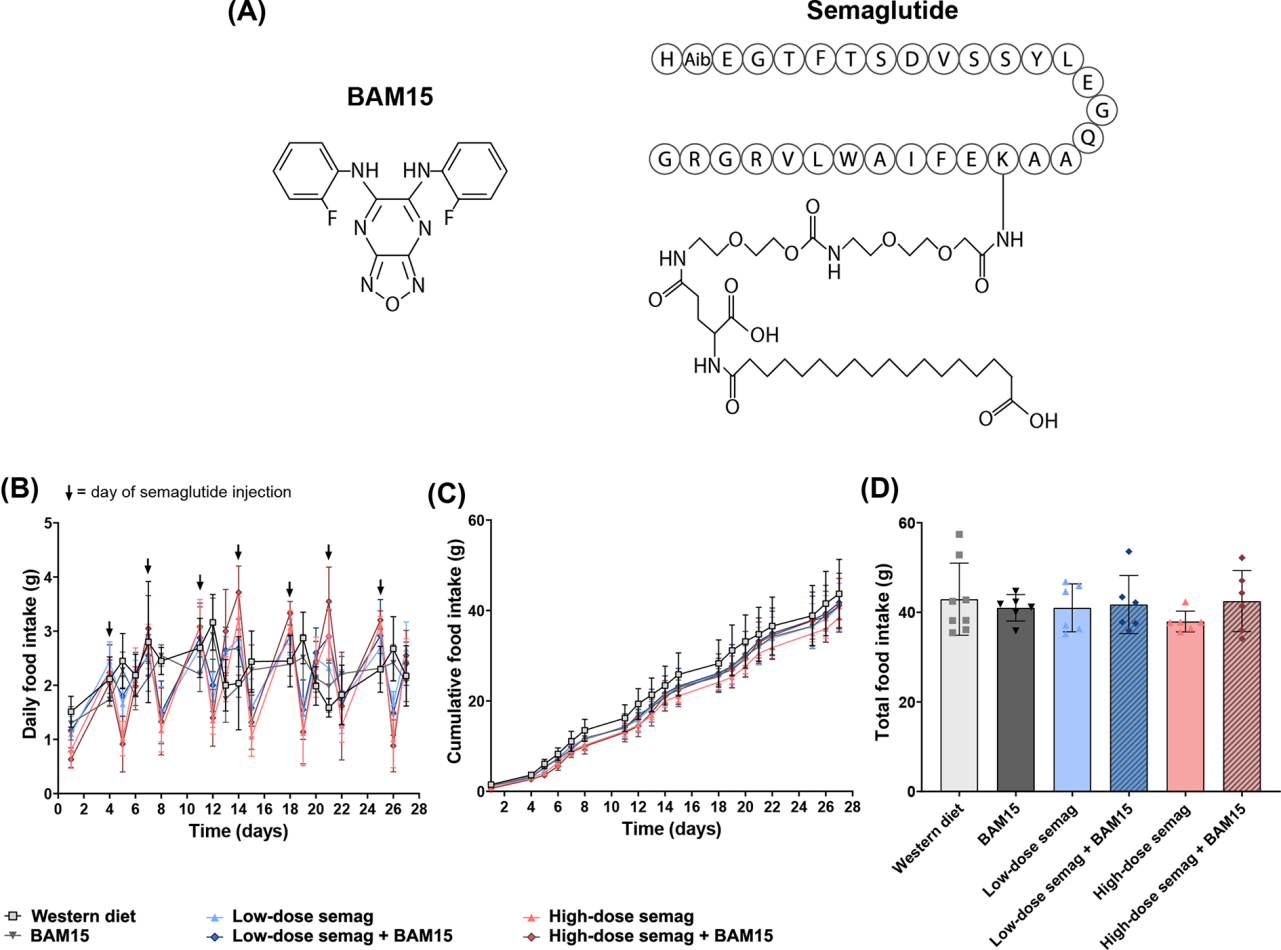
Semaglutide and BAM15 did not alter total food intake Chemical structures of BAM15 and semaglutide (**A**). Letters in circles indicate one-letter amino acid code for peptide section of semaglutide. Aib = aminoisobutyric acid. Daily food intake (**B**), cumulative food intake (**C**), and total food intake (**D**) in mice treated with BAM15 and/or semaglutide. Semag = semaglutide. Graphs show mean ± SD; *n* = 6–8.

Mice fed western diet (WD) for 5 weeks were stratified into six groups and treated as follows for 4 weeks: no-drug WD control, BAM15, low-dose (5 µg/kg) semaglutide, low-dose semaglutide + BAM15, high-dose (20 µg/kg) semaglutide, and high-dose semaglutide + BAM15. The chemical structures of BAM15 and semaglutide are shown in Figure 1A. Food intake measurements confirmed that semaglutide was exerting the expected effects to temporarily suppress appetite, consistent with its mechanism of action to increase satiety. At both doses, semaglutide caused hypophagia for ∼24 h following injection (Figure 1B). However, this was followed by 1–2 days of a compensatory increase in food intake after the acute effects of semaglutide injection had subsided (Figure 1B). Consequently, the overall cumulative food intake and total food intake were not significantly different among any treatment groups (Figure 1C,D).

**Figure 2:**
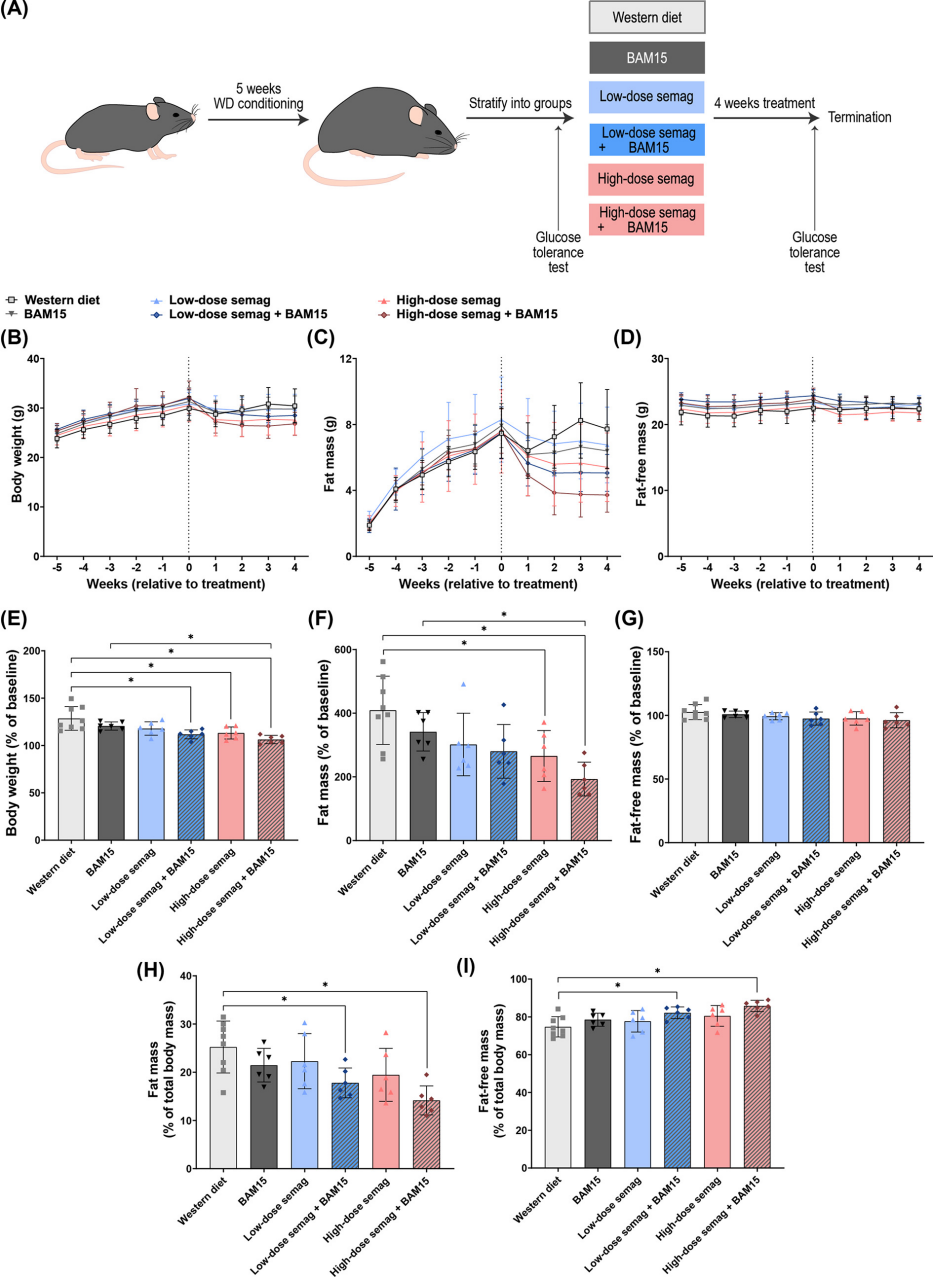
High-dose semaglutide combined with BAM15 decreased body weight and body fat composition Schematic diagram of treatment regime (**A**). Weekly body weight during WD conditioning (before dotted line) and treatment period (after dotted line) (**B**). Weekly monitoring of fat mass (**C**) and fat-free mass (**D**). Body weight (**E**), fat mass (**F**), and fat-free mass (**G**) after 4 weeks of treatment as a percentage of baseline (week 5) values. Percentage fat mass (**H**) and fat-free mass (**I**) over total body mass after 4 weeks of treatment. Semag = semaglutide. * indicates *P*<0.05 by one-way ANOVA with Sidak’s multiple comparisons test. Graphs show mean ± SD; *n* = 6–8.

Body weight, fat mass, and fat-free lean mass were measured over the course of the study period (illustrated in Figure 2A) and are shown longitudinally as raw weight change in Figure 2B–D. However, because mice started this study with differences in baseline body weight, it is more appropriate to express the data normalised as percent change from baseline for each animal (Figure 2E–G). The combination of BAM15 with high-dose semaglutide had the greatest weight loss effects and resulted in the lowest average body weight as a percentage change from baseline values (week 5, before conditioning) (106 ± 4%), which was significantly different compared with both WD (128 ± 13%, *P*<0.0001) and BAM15 monotherapy (120 ± 4%, *P*=0.024) (Figure 2E). When normalised to baseline, body weight was also significantly decreased in mice given high-dose semaglutide alone (113 ± 6%, *P*=0.0067) (Figure 2E). While neither BAM15 nor low-dose semaglutide alone were able to significantly decrease body weight, mice given the combination of BAM15 and low-dose semaglutide had lower normalised body weight (111 ± 5%) compared with WD controls (*P*=0.0024) (Figure 2E). The combination of BAM15 and high-dose semaglutide also had the greatest effects on fat mass and resulted in the lowest average fat mass as a percentage of baseline (193 ± 53%), which was significantly different compared with both WD (409 ± 107%, *P*=0.0004) and BAM15 alone (342 ± 60%, *P*=0.041) (Figure 2F). High-dose semaglutide alone also decreased fat mass normalised to baseline (266 ± 80%, *P*=0.032), while the remaining treatments resulted in trends for decreased fat mass (Figure 2F). Fat-free lean mass was not significantly changed by any of the treatments (Figure 2G).

EchoMRI data showed that body weight losses caused by BAM15 and semaglutide were primarily due to changes in fat mass. The greatest improvement in body composition was observed in mice that received BAM15 combined with high-dose semaglutide, which had significantly decreased percentage fat mass over total body mass (14.2 ± 3.0%) compared with WD controls (25.2 ± 5.4%, *P*=0.0008) (Figure 2H). The combination of BAM15 with low-dose semaglutide also significantly decreased percentage fat mass (17.8 ± 3.1%) compared with WD controls (*P*=0.045), which was a better result than high-dose semaglutide monotherapy (19.5 ± 5.5%) (Figure 2H). Fat-free lean mass as a percentage of total body mass was significantly increased by both combination treatments of low- or high-dose semaglutide and BAM15, compared with the WD group (Figure 2I).

**Figure 3:**
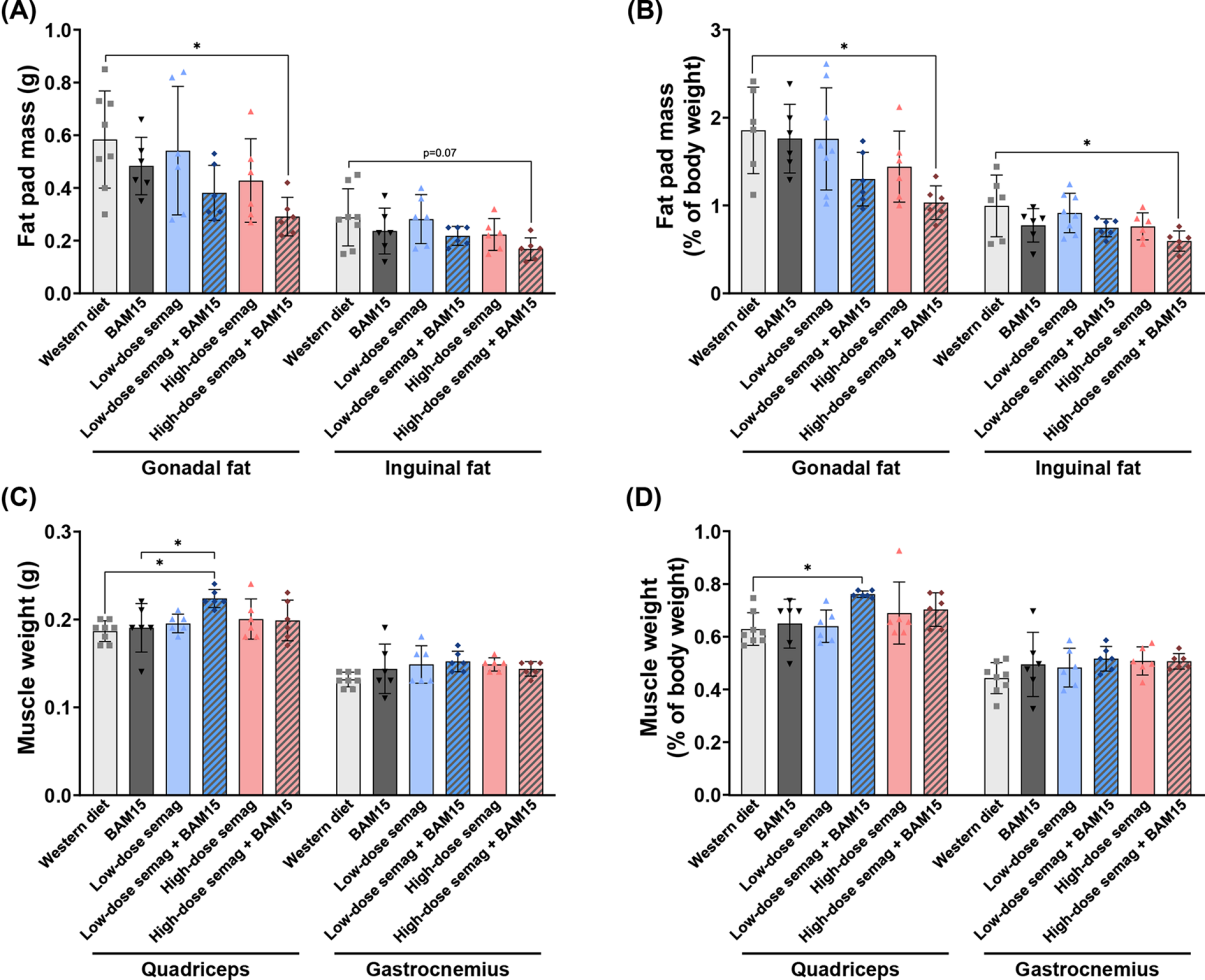
Tissue wet weights at termination Wet weights of single gonadal and inguinal fat pads (**A**), fat pad weights normalised to body weight (**B**), wet weights of quadriceps and gastrocnemius muscles (**C**), muscle weights normalised to body weight (**D**). Semag = semaglutide. * indicates *P*<0.05 by one-way ANOVA with Sidak’s multiple comparisons test. Graphs show mean ± SD; *n*=6–8.

To more directly interrogate body composition, wet weights of adipose and muscle tissues were measured at termination. Compared with WD controls, the combination of high-dose semaglutide and BAM15 significantly decreased gonadal fat pad mass (0.29 ± 0.07 vs. 0.58 ± 0.18 g, *P*=0.016) and resulted in a trend for decreased inguinal fat pad mass (0.17 ± 0.04 vs. 0.29 ± 0.11 g, *P*=0.07, Figure 3A). In contrast, the groups given BAM15, high-dose semaglutide, and BAM15 + low-dose semaglutide showed trends for decreased fat pad mass that did not reach statistical significance (Figure 3A). Due to variation among animal body weights, fat pad masses were also expressed as a percentage of body weight (Figure 3B). The greatest fat-lowering effects were achieved by high-dose semaglutide combined with BAM15. As a percentage of body weight, the combination of high-dose semaglutide and BAM15 decreased gonadal fat pad weight by 47% (1.0 ± 0.2 vs. 1.9 ± 0.5%, *P*=0.003) and inguinal fat pad weight by 38% (0.6 ± 0.1 vs. 1.0 ± 0.3%, *P*=0.028) compared with WD (Figure 3B). Low-dose semaglutide combined with BAM15 showed a non-significant trend toward decreasing gonadal fat pad mass (1.3 ± 0.3%, *P*=0.07) as a percentage of body weight (Figure 3B). The wet weights of the quadriceps and gastrocnemius muscles were not decreased by any drug treatment. However, quadriceps weight was unexpectedly increased in mice given the combination of low-dose semaglutide and BAM15 (0.22 ± 0.01 g), compared with WD (0.19 ± 0.01 g, *P*=0.008) and BAM15 alone (0.19 ± 0.03 g, *P*=0.036), but gastrocnemius weights were unchanged among treatments (Figure 3C). When normalized to body weight, quadriceps weight remained significantly higher in the group given low-dose semaglutide combined with BAM15 (0.77 ± 0.01%) compared with WD controls (0.63 ± 0.06%, *P*=0.022) (Figure 3D), which is consistent with the increased percentage fat-free lean mass that was observed in this group through EchoMRI at all time points during the study (Figure 2D,I).

**Figure 4:**
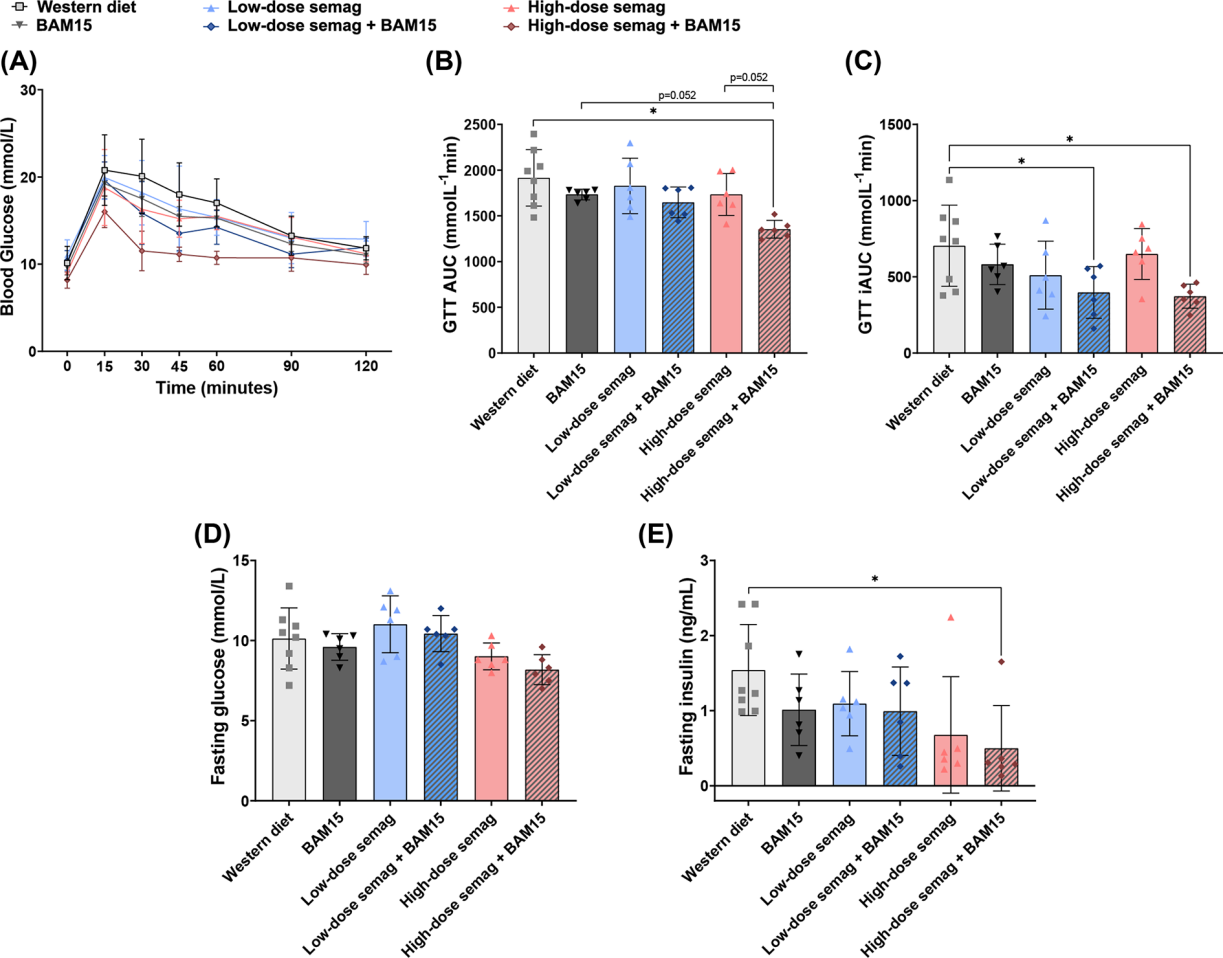
High-dose semaglutide combined with BAM15 caused the greatest improvement in glucose homeostasis Glucose tolerance curves (**A**), total area under the curve (**B**), and incremental area under the curve (**C**) after 4 weeks of treatment. Fasting glucose levels (**D**) and fasting insulin levels (**E**) after 4 weeks of treatment. Semag = semaglutide. * indicates *P*<0.05 by one-way ANOVA with Sidak’s multiple comparisons test (**B–E**). Graphs show mean ± SD; *n*=6–8.

Glucose tolerance tests (GTTs) were performed to assess blood glucose handling in drug-treated mice compared with WD controls. The combination treatment of high-dose semaglutide and BAM15 achieved better effects on glucose tolerance than all other treatments; this group had the lowest blood glucose levels during the GTT (Figure 4A) and significantly decreased total AUC (1353 ± 97 mmolL^−1^·min) compared with WD controls (1917 ± 309 mmolL^−1^·min, *P*=0.0005) (Figure 4B). The mice given high-dose semaglutide combined with BAM15 also showed a trend for decreased AUC compared with BAM15 alone (1734 ± 58 mmolL^−1^·min, *P*=0.052) or high-dose semaglutide alone (1734 ± 230 mmolL^−1^·min, *P*=0.052) (Figure 4B). To account for heterogeneity in baseline blood glucose, incremental area under the curve (iAUC) was also calculated. Both combination treatments significantly decreased iAUC compared with WD controls (704 ± 266 mmolL^−1^·min vs. 398 ± 170 mmolL^−1^·min for low-dose semaglutide and BAM15 [*P*=0.046], and 373 ± 79 mmolL^−1^·min for high-dose semaglutide and BAM15 [*P*=0.025], Figure 4C). However, none of the monotherapies significantly changed iAUC (Figure 4C). Fasting glucose was not significantly changed among the treatment groups (Figure 4D), but fasting insulin levels were significantly decreased by 68% in the group given high-dose semaglutide combined with BAM15 (0.50 ± 0.57 ng/ml), compared with the WD control (1.54 ± 0.60 ng/ml, *P*=0.022) (Figure 4E).

**Figure 5:**
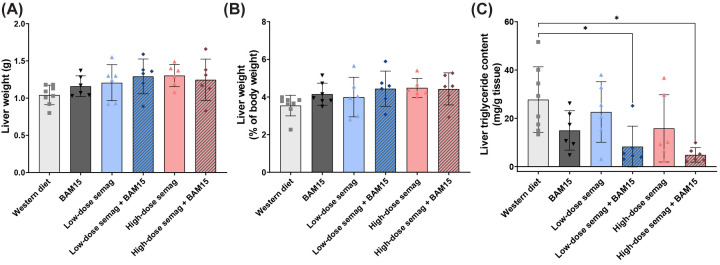
Liver triglyceride content was decreased by combination treatments of BAM15 and semaglutide Liver wet weights as raw values (**A**) and normalized to body weight (**B**). Liver triglyceride content in frozen liver tissue (**C**). Semag = semaglutide. * indicates *P*<0.05 by one-way ANOVA with Sidak’s multiple comparisons test. Graphs show mean ± SD; *n*=6–8.

To investigate the effects of the drug treatments on liver fat, liver wet weights were recorded at termination and triglyceride content was measured in frozen liver tissue. Liver wet weights were not significantly changed among the treatment groups (Figure 5A,B). Monotherapy with BAM15 and both doses of semaglutide resulted in trends for decreased liver triglyceride content that did not reach statistical significance (Figure 5C). However, liver triglyceride content was decreased by 70% and 82% in mice given the combination of BAM15 with low-dose (8.3 ± 8.4 mg/g, *P*=0.0042) or high-dose semaglutide (4.9 ± 3.0 mg/g, *P*=0.021), respectively, compared with WD controls (27.7 ± 13.6 mg/g) (Figure 5C). To investigate potential drug-induced changes in fatty acid synthesis, Western blotting was performed to assess the protein expression and phosphorylation state of key enzymes. However, there were no significant changes in protein expression among the groups for total or phosphorylated acetyl-CoA carboxylase (ACC) relative to total ACC; ACC catalyses the formation of malonyl-CoA (Supplementary Figure S1A,B). Similarly, no significant changes were observed in levels of fatty acid synthase (FAS), which uses malonyl-CoA as a substrate for *de novo* fatty acid production (Supplementary Figure S1A,C). AMPK is the upstream kinase that inactivates ACC by phosphorylation. Consistent with ACC phosphorylation results, there were no significant changes in the expression of phosphorylated AMP-dependent kinase (AMPK) relative to total AMPK (Supplementary Figure S1A,D). To investigate potential drug-induced changes in fatty acid oxidation, the activities of key fat oxidation enzymes medium-chain acyl-CoA dehydrogenase (MCAD) and 3-hydroxyacyl-CoA dehydrogenase (β-HAD) were measured from frozen liver tissue lysates. However, there were no significant changes in enzyme activity among the treatment groups (Supplementary Figure S1E,F).

We next investigated the effects of BAM15 and semaglutide treatment on energy expenditure and respiratory control ratio by indirect calorimetry in a separate cohort of mice fed WD, BAM15, high-dose semaglutide, or a combination of BAM15 and high-dose semaglutide. Mice were fed WD for 2 weeks, then 24 h of baseline measurements were taken to assess oxygen consumption, carbon dioxide output, and locomotor activity. Mice were then either maintained on WD or switched to WD containing 0.05% BAM15. After a further 24 h, mice were injected subcutaneously with either high-dose semaglutide (20 µg/kg) or vehicle as indicated in Supplementary Figure S2. The submaximal dose of BAM15 used in the present study, slowly consumed in the food over time, was insufficient to generate any detectable changes in the rate of oxygen consumption or carbon dioxide output (Supplementary Figure S2A–F); however, a previous study has shown that an acute oral gavage of a similar dose to the total that is eaten over 24 h (50 mg/kg) increases oxygen consumption acutely for 1 h post-dose (*P*=0.05) [43]. Thus, the indirect calorimeter is not sensitive enough to detect small increases in oxygen consumption rate in freely moving mice consuming drug *ad libitum*. Similarly, high-dose semaglutide did not significantly alter oxygen consumption (Supplementary Figure S2A,B). However, we did observe that high-dose semaglutide, given either alone or combined with BAM15, caused an acute decrease in the respiratory exchange ratio (RER, ratio of *V*CO_2_/*V*O_2_) compared with WD, which indicates a shift toward increased lipid oxidation (Supplementary Figure S2E,F). This decrease in RER was driven primarily by decreased *V*CO_2_, which was statistically lower in mice given high-dose semaglutide + BAM15 compared with WD (Supplementary Figure S2D). However, locomotor activity was also significantly decreased by high-dose semaglutide compared with WD controls (Supplementary Figure S2G,H), suggesting that this dose of semaglutide caused a shift from carbohydrate oxidation to fat oxidation in conjunction with decreased physical activity.

## Discussion

GLP-1-RA drugs have rapidly increased in popularity and become widely used for weight loss and blood glucose management. The selection of available GLP-1-RAs continues to expand, with the addition of tirzepatide, a dual agonist of GLP-1 receptor and glucose-dependent insulinotropic polypeptide (GIP) receptor [60]. There is also a movement towards using higher doses of GLP-1-RAs, with the goal of achieving greater weight loss and glucose-lowering efficacy in a wider selection of patients. The approved doses of GLP-1-RA drugs semaglutide and dulaglutide have both increased since they first entered the market [13,61], and recent trial results support the use of higher tirzepatide doses to achieve maximal efficacy [62–64]. However, increasing doses of GLP-1-RAs are associated with greater frequency of adverse effects, especially gastrointestinal side effects [20,64].

GLP-1-RAs primarily function by decreasing calorie intake, but chronic usage results in a plateau in weight loss due to compensatory effects of the body to increase metabolic efficiency that hinders further weight loss [65]. Herein, we investigated a novel strategy to overcome the body’s compensatory mechanisms of weight preservation by combining the GLP-1-RA semaglutide with a mitochondrial uncoupler BAM15. Mitochondrial uncouplers act via a distinct mechanism of action to GLP-1-RAs by decreasing metabolic efficiency without affecting food intake [38–41,43,44,46,47]. Therefore, we hypothesized that combining a GLP-1-RA with a mitochondrial uncoupler may achieve additive effects on weight loss and glucose-lowering efficacy at low doses to minimize adverse effects. However, it was uncertain whether the combination treatment would be sufficient to overcome the compensatory mechanisms that defend the body against weight loss, and whether BAM15 would counteract the anorexic effects of semaglutide by increasing food intake, similar to the effects of exercise [6,7]. Ultimately this study showed that semaglutide and BAM15 had beneficial effects when used together that were not achievable by either monotherapy alone. Specifically, combining the higher dose of 20 µg/kg semaglutide with BAM15 admixed in food at 0.05% (w/w) achieved the greatest metabolic outcomes in this study, not only decreasing body weight and adiposity but also improving glucose tolerance and liver triglyceride content compared with vehicle control. In contrast, most of these effects could not be achieved by either high-dose semaglutide or BAM15 alone.

Importantly, the weight loss effects produced by semaglutide combined with BAM15 were driven by fat mass loss while preserving lean muscle mass. Notably, body fat percentage was significantly decreased only in the two combination treatment groups that received BAM15 combined with either dose of semaglutide. Consistent with EchoMRI data, mice given high-dose semaglutide combined with BAM15 had decreased fat pad weights measured at termination. In contrast, none of the drug treatments decreased the wet weights of quadriceps or gastrocnemius muscle. In fact, the low-dose semaglutide + BAM15 group had increased quadriceps weight compared with WD control; these animals started the study with higher lean mass, which appeared to be preserved throughout the drug treatment.

Food intake was not affected by BAM15 admixed in WD at 0.05% (w/w). In mice that were administered semaglutide, hypophagia was observed when food intake was measured the next day, consistent with the mechanism of action of semaglutide to enhance satiety. However, compensatory over-eating in the following days leading up to the next dose of semaglutide resulted in no overall change in total food intake over the entire treatment period. Therefore, the weight loss and glucose tolerance benefits of these drugs were independent of a detectable change in overall calorie intake. Nevertheless, it is possible that the periods of reduced food intake in between bouts of compensatory overeating in semaglutide-treated mice may have conferred some benefit similar to that of intermittent fasting [66]. The pseudo-intermittent fasting effect may also have contributed to the decreased fat pad masses that were observed in the group treated with the combination of high-dose semaglutide and BAM15, as intermittent fasting has previously been shown to induce adipose browning in mice [67]. In the present study, semaglutide treatment did not affect cumulative food intake, which is likely due to the considerably lower doses used when compared with other groups [49,68].

The combination of high-dose semaglutide and BAM15 was the only treatment to result in significantly improved glucose tolerance and decreased plasma insulin levels relative to WD controls. The lower dose of semaglutide combined with BAM15 also improved glucose tolerance, although it did not significantly impact insulin secretion. Both BAM15 and semaglutide are known to exert glucose-lowering effects in mice and/or humans [43,50,55], and their combination likely achieves improved effects through improving insulin sensitivity in diet-induced obese mice. Future work is required to investigate the mechanism of improved glucose metabolism with the combination treatment by assessing insulin sensitivity in skeletal muscle, liver, adipose, and/or brain. In addition, oral glucose tolerance testing could provide additional information as oral glucose has stronger effects on insulin secretion than i.p. delivered glucose.

BAM15 in combination with either dose of semaglutide also had stronger effects to lower liver triglyceride levels than BAM15 or either dose of semaglutide alone. The beneficial effects of BAM15 on liver steatosis have been previously published in diet-induced obese mice at a 2-fold higher dose of 0.1% admixed in diet [43], and in *db/db* mice at a 4-fold higher dose of 0.2% admixed in diet [47]. Decreased liver fat content in BAM15-treated mice is consistent with the mitochondrial uncoupling mechanism of BAM15, which necessitates increased nutrient oxidation, including fatty acid oxidation. While we were unable to detect changes across groups in liver β-oxidation enzyme activity, this may be due to the time of tissue harvest, as tissues were collected at the start of the light cycle, >12 h after the bulk of feeding activity at the beginning of the dark cycle [43]. At this time, BAM15 levels are relatively low [43] and there would be minimal nutrient-related signaling that might be expected to induce changes in fat metabolism. In contrast, radiotracer experiments previously showed that BAM15 increases ^14^C-palmitate oxidation in liver tissue homogenates after an acute oral dose of 100 mg/kg [43], ∼2× higher than the total dose received per mouse per day in the present study. Therefore, we cannot rule out that subtle changes in fatty acid oxidation enzyme activity may exist but are below our sensitivity of detection due to a combination of low BAM15 dose and timing of euthanasia. Semaglutide has also been shown to have advantageous effects on fatty liver in preclinical mouse data [69]. It has been proposed that the anti-hepatosteatotic effects of semaglutide relate to increased liver expression of genes relating to fatty acid oxidation and decreased expression of genes implicated in triglyceride synthesis [52,55]. Hence, the liver triglyceride-lowering effects of the combined semaglutide and BAM15 treatments are likely related subtle changes in fatty acid oxidation, but further work is necessary to detect such changes. Similarly, there may also be differences in hepatic lipogenesis. We were unable to detect differences in protein expression levels of fatty acid synthase (FAS) or phosphorylated acetyl-CoA carboxylase (ACC), but we cannot exclude the possibility of subtle effects that were not distinguishable by Western blotting, or effects that would be observable only in the immediate post-prandial state or fasting state.

The human body has strong compensatory mechanisms that defend against weight loss. In response to conventional diet and exercise therapy, patients have been observed to exhibit improved metabolic efficiency, increased appetite, and decreased non-exercise activity [5,6,27–29,70], all of which limit the beneficial metabolic outcomes of their treatment. In the present study, the combination of semaglutide and BAM15 was not observed to cause any compensatory increase in overall food intake, though all semaglutide-treated mice showed an expected altered feeding pattern with periods of suppressed food intake followed by overeating. The short-term suppression of food intake was consistent with indirect calorimetry results showing that semaglutide caused a decrease in physical activity and a shift toward fat oxidation as indicated by a decreased RER. Importantly, however, the phenotyping results show that combining semaglutide and BAM15 achieved effects of a sufficient magnitude that potential compensatory mechanisms did not entirely mitigate the drug effects, thus demonstrating the feasibility of this therapeutic approach. In future, an informative experiment would be to compare these combination treatments with a maximally effective dose of semaglutide to determine whether it is possible to achieve comparable metabolic outcomes.

A limitation of the present study was that only male mice were used. Future studies should investigate whether the effects of semaglutide combined with BAM15 are reproducible in female mice. In addition, a potential future direction is to explore newer GLP-1-RA molecules, such as tirzepatide, which also acts on the glucose-dependent insulinotropic polypeptide (GIP) receptor and has shown stronger weight loss and HbA1c-lowering effects than semaglutide at the doses tested in the SURPASS-2 trial [62]. Tirzepatide results in similar gastrointestinal side effects as semaglutide, including nausea, vomiting, and diarrhoea [62,64]; therefore, it may also be beneficial to combine tirzepatide with an uncoupler to permit a lower dose of tirzepatide to be used.

Overall, we have shown that combining submaximal doses of semaglutide and the mitochondrial uncoupler BAM15 resulted in stronger metabolic benefits than either drug alone compared with vehicle control including body weight loss, decreased fat mass, improved glucose tolerance, and decreased liver triglyceride content. Semaglutide and BAM15 monotherapies trended to have partial metabolic improvements compared with the combination treatment; therefore, the combination groups were significantly different from vehicle but not from either monotherapy group. This study demonstrates the efficacy of a new therapeutic strategy to simultaneously decrease energy intake and increase energy expenditure through pharmacological means, which may permit the use of lower doses of drug while still achieving maximal benefit to improve metabolic outcomes.

## Clinical perspectives

- Glucagon-like peptide 1 receptor agonists (GLP-1-RAs) such as semaglutide have strong weight loss and glucose-lowering effects but require dose escalation that may increase risk of adverse side effects.
- This study showed that combining semaglutide with the mitochondrial uncoupler BAM15 had weight loss, anti-steatosis, and glucose-lowering effects in diet-induced obese mice.
- Combining GLP-1-RAs with a mitochondrial uncoupler may represent a novel approach to achieve optimal metabolic outcomes using low doses of drugs to minimize adverse effects.

## Supplementary Material

Supplementary Figures S1-S2

## Data Availability

Data are available upon reasonable request to the corresponding author.

## Abbreviations

β-HAD: 3‐hydroxyacyl‐CoA dehydrogenase
ACC: acetyl-CoA carboxylase
AMPK: AMP-dependent kinase
DNP: 2,4-dinitrophenol
FAS: fatty acid synthase
GIP: glucose-dependent insulinotropic polypeptide
GLP-1-RA: glucagon-like peptide 1 receptor agonist
GTT: glucose tolerance test
iAUC: incremental area under the curve
MCAD: medium chain acyl-CoA dehydrogenase
RER: respiratory exchange ratio
