# Supplementary material for: Beneficial effects of simultaneously targeting calorie intake and calorie efficiency in diet-induced obese mice
Source: Clin Sci (Lond). 2024 Feb 19;138(4):173–87. doi: 10.1042/CS20231016 (PMC10876416; doi:10.1042/CS20231016)
Supplement: Supplementary Figures S1-S2 [file CS-2023-1016_supp.pdf]

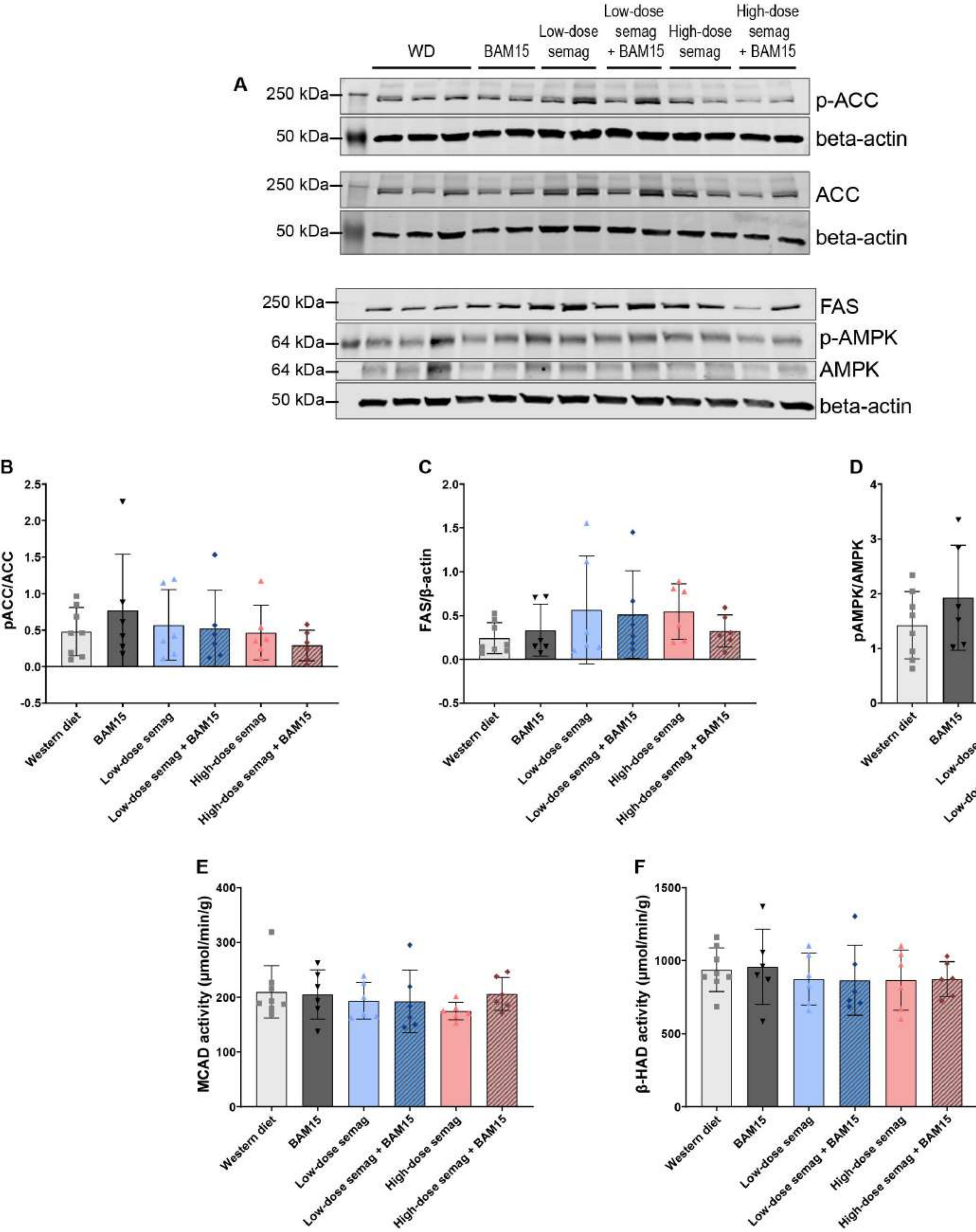

A
WD
BAM15
Low-dose semag
Low-dose semag + BAM15
High-dose semag
High-dose semag + BAM15
250 kDa
50 kDa
64 kDa
p-ACC
beta-actin
ACC
FAS
p-AMPK
AMPK
B
pACC/ACC
C
FAS/β-actin
D
pAMPK/AMPK
E
MCAD activity (µmol/min/g)
F
β-HAD activity (µmol/min/g)
Western diet
BAM15
Low-dose semag
Low-dose semag + BAM15
High-dose semag
High-dose semag + BAM15

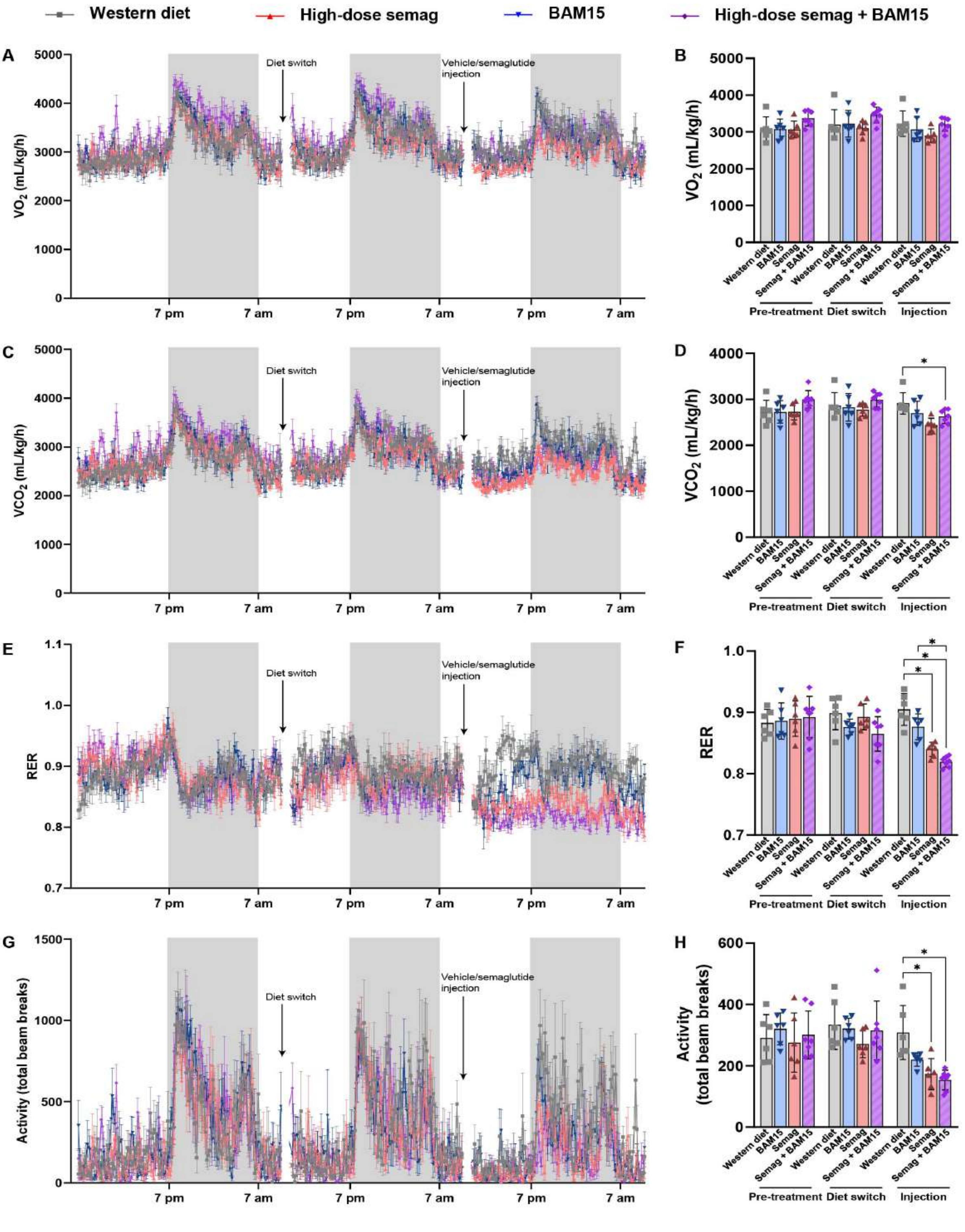

Western diet
High-dose semag
BAM15
High-dose semag + BAM15
A
VO2 (mL/kg/h)
Diet switch
Vehicle/semaglutide injection
7 pm
7 am
B
Western diet
BAM15
Semag
Semag + BAM15
Pre-treatment
Diet switch
Injection
C
VCO2 (mL/kg/h)
D
E
RER
F
G
Activity (total beam breaks)
H

Beneficial effects of simultaneously targeting calorie intake and calorie efficiency in diet-induced obese mice

Sing-Young Chen et al.

Supplemental Material

Western blot full gels

Gel 1a: ACC & B-Actin

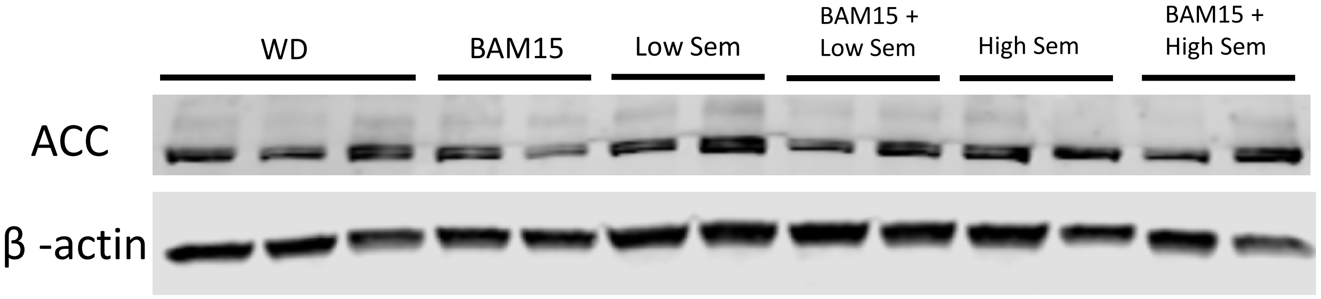

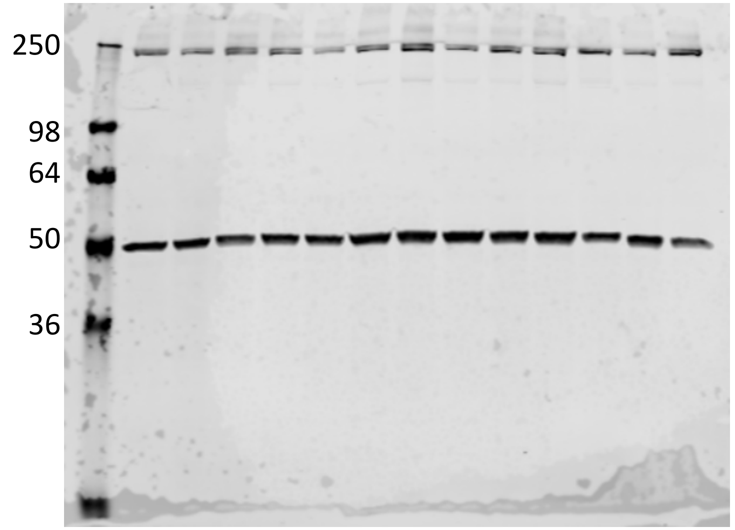

Groups:

1. WD (mice 1-3)
2. HFD + BAM15 (mice 1-2)
3. HFD + Low Semaglutide (mice 1-2)
4. HFD + Low Semaglutide + BAM15 (mice 1-2)
5. HFD + High Semaglutide (mice 1-2)
6. HFD + High Semaglutide + BAM15 (mice 1-2)

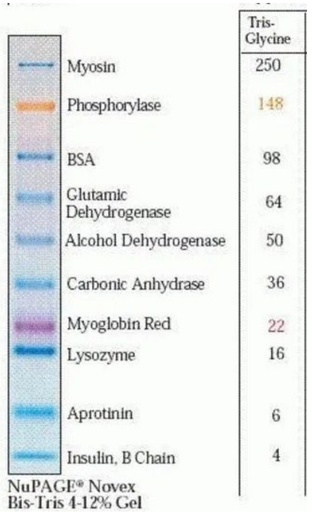

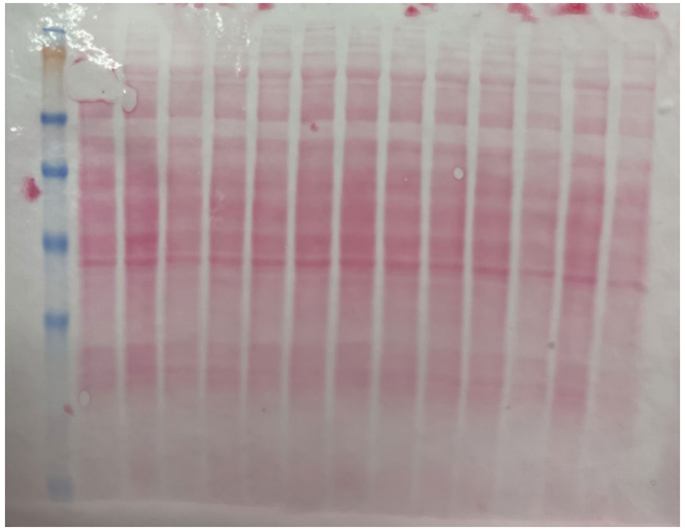

Gel 1b: ACC & B-Actin

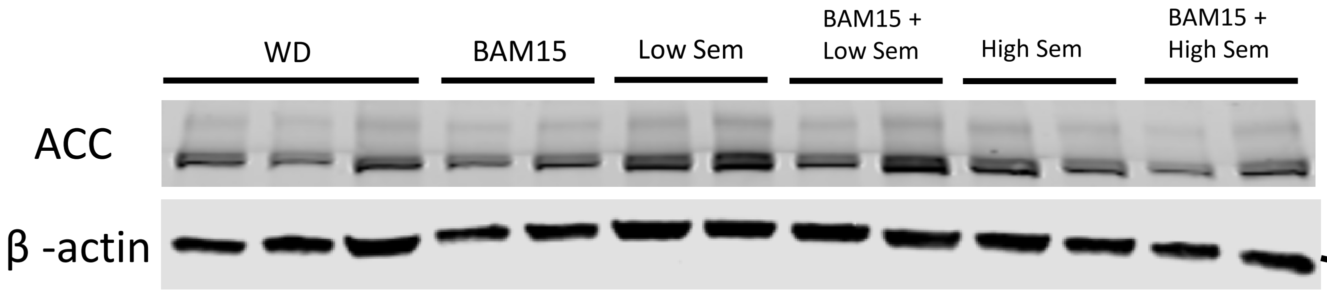

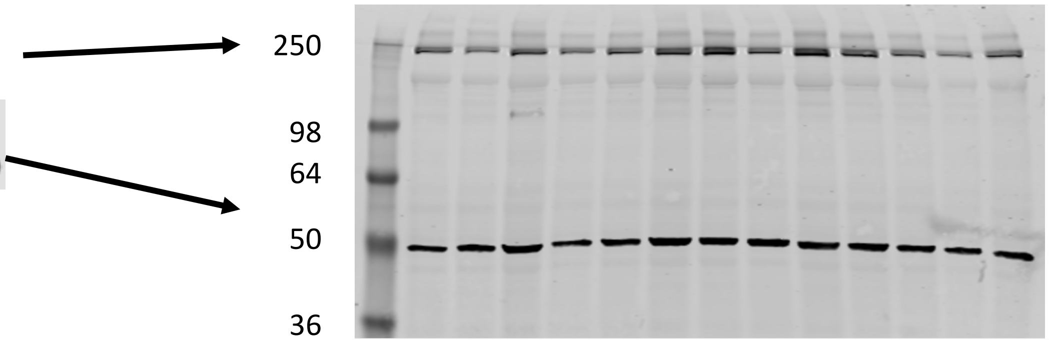

Groups:

1. WD (mice 4-6)
2. HFD + BAM15 (mice 3-4)
3. HFD + Low Semaglutide (mice 3-4)
4. HFD + Low Semaglutide + BAM15 (mice 3-4)
5. HFD + High Semaglutide (mice 3-4)
6. HFD + High Semaglutide + BAM15 (mice 3-4)

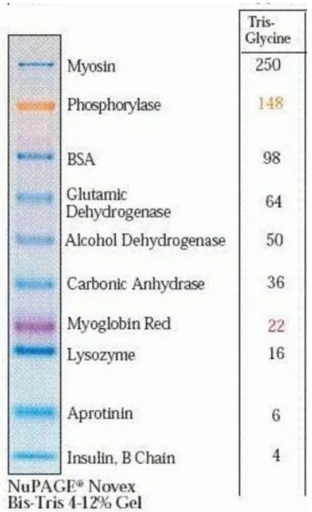

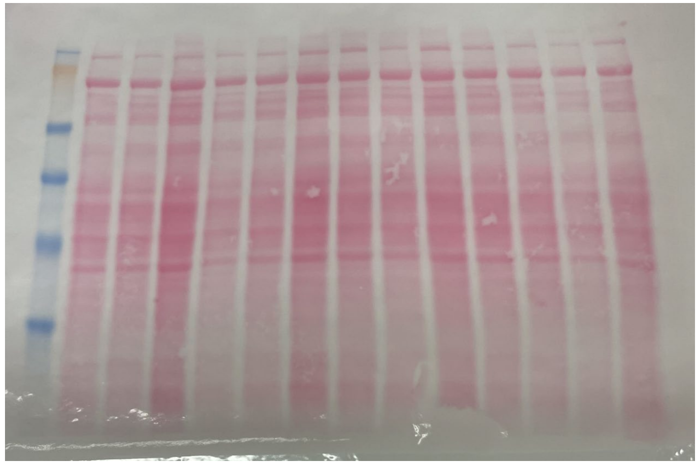

Gel 1c: ACC & B-Actin

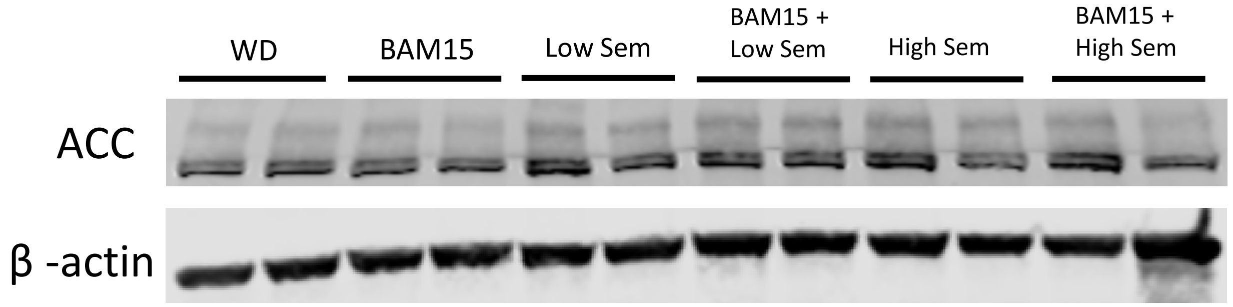

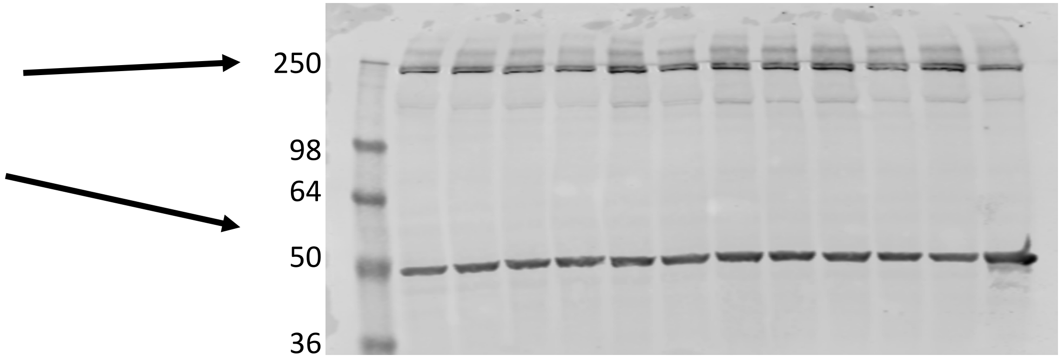

Groups:

1. WD (mice 7-8)
2. HFD + BAM15 (mice 5-6)
3. HFD + Low Semaglutide (mice 5-6)
4. HFD + Low Semaglutide + BAM15 (mice 5-6)
5. HFD + High Semaglutide (mice 5-6)
6. HFD + High Semaglutide + BAM15 (mice 5-6)

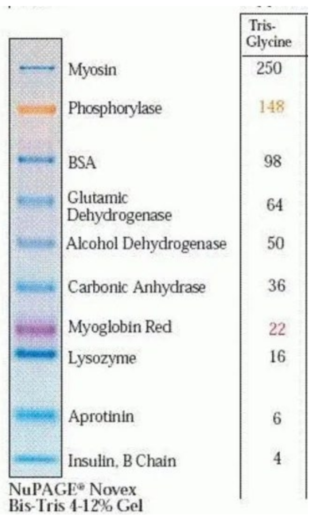

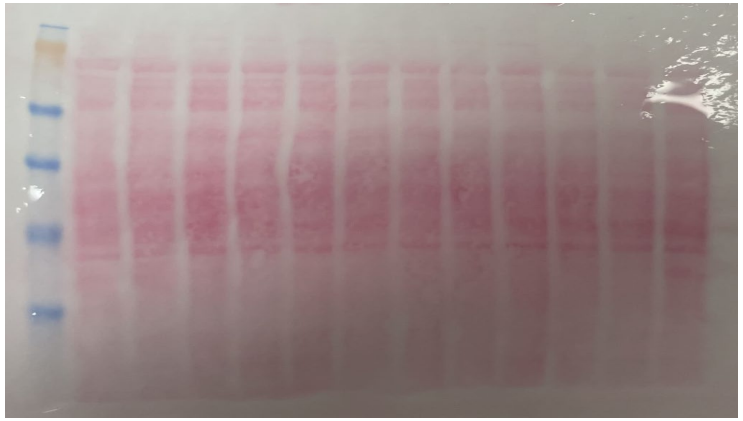

Gel 2a: pACC & B-Actin

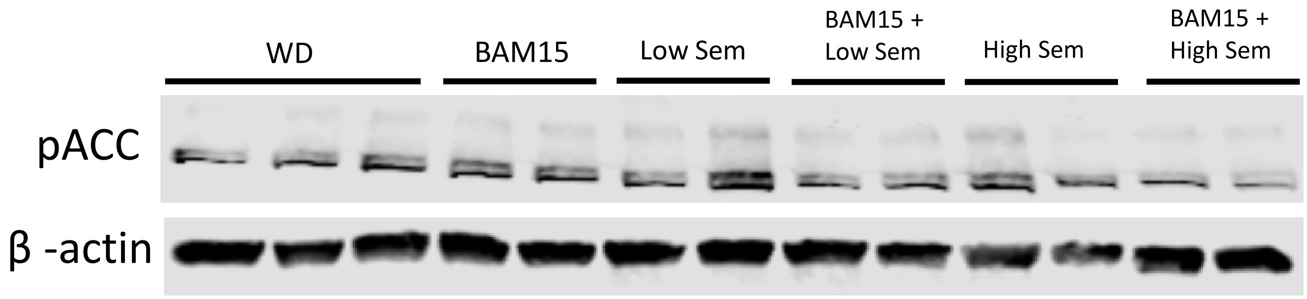

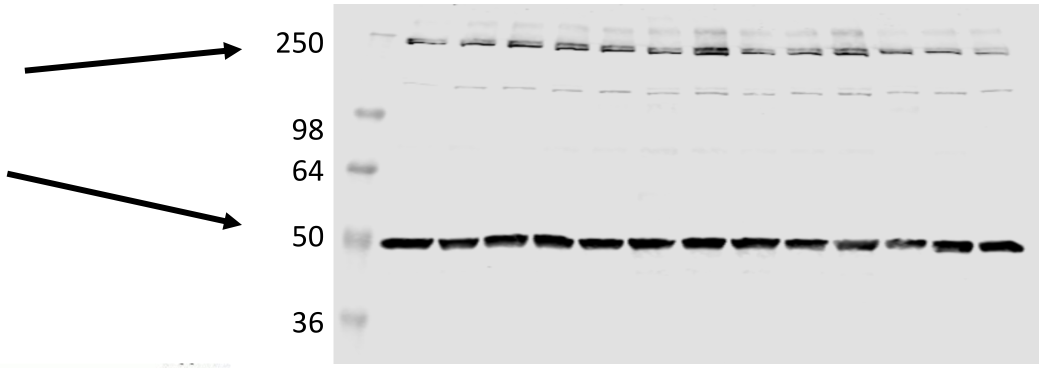

Groups:

1. WD (mice 1-3)
2. HFD + BAM15 (mice 1-2)
3. HFD + Low Semaglutide (mice 1-2)
4. HFD + Low Semaglutide + BAM15 (mice 1-2)
5. HFD + High Semaglutide (mice 1-2)
6. HFD + High Semaglutide + BAM15 (mice 1-2)

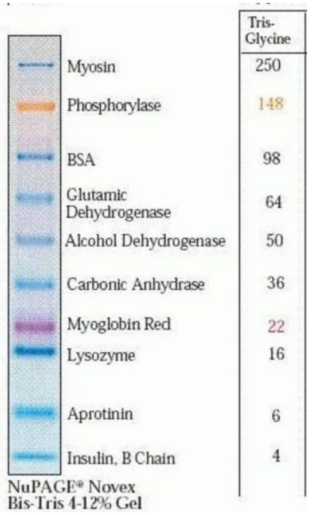

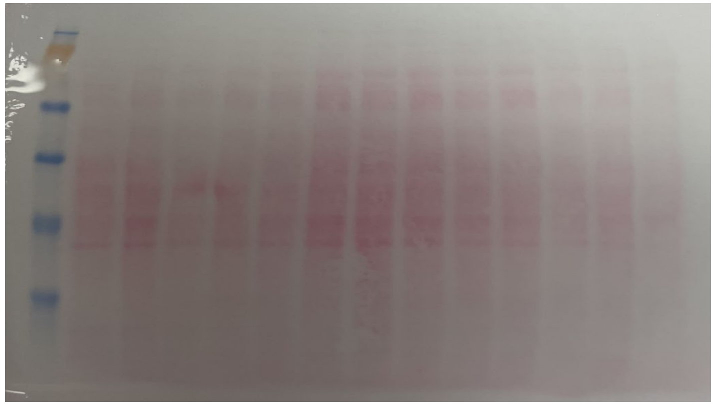

Gel 2b: pACC & B-Actin

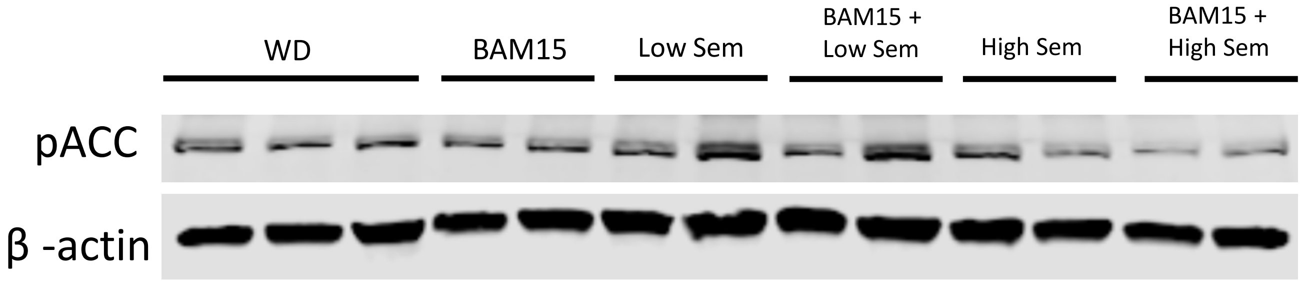

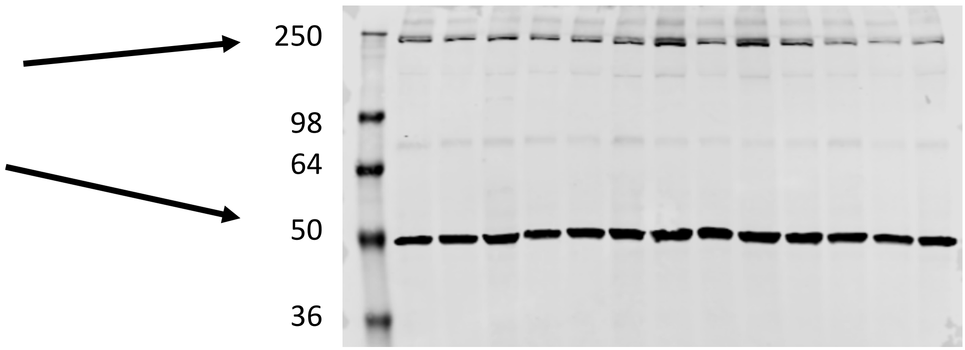

Groups:

1. WD (mice 4-6)
2. HFD + BAM15 (mice 3-4)
3. HFD + Low Semaglutide (mice 3-4)
4. HFD + Low Semaglutide + BAM15 (mice 3-4)
5. HFD + High Semaglutide (mice 3-4)
6. HFD + High Semaglutide + BAM15 (mice 3-4)

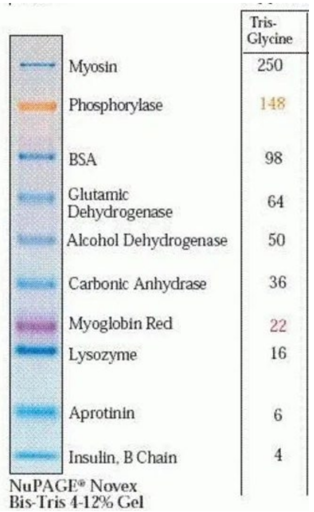

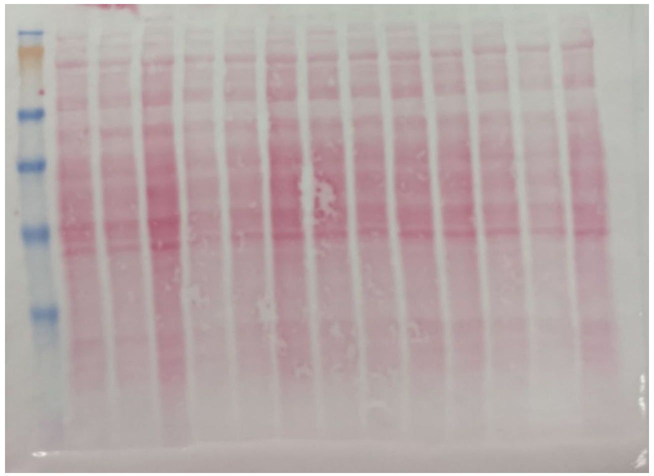

Gel 2c: pACC & B-Actin

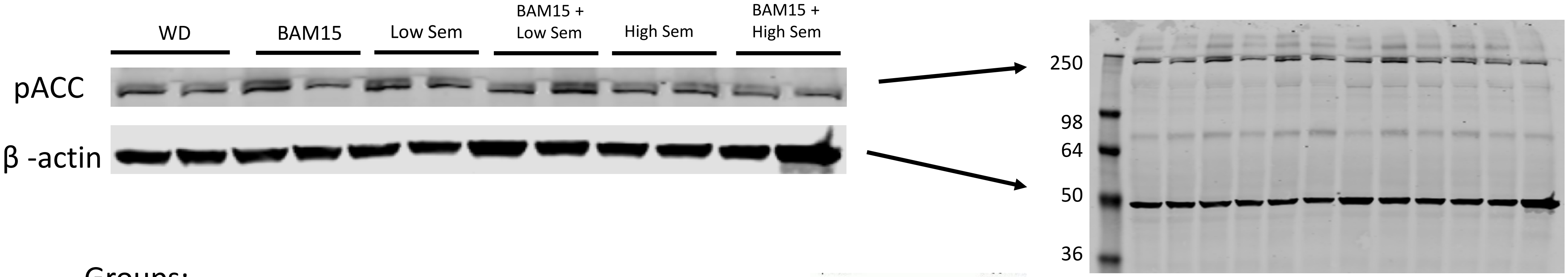

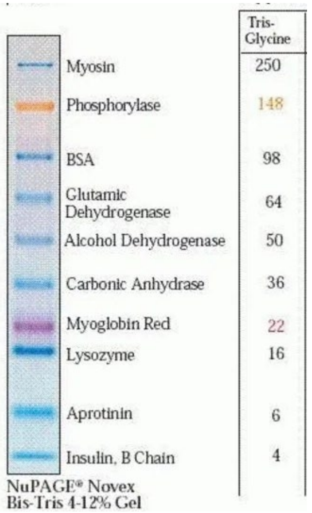

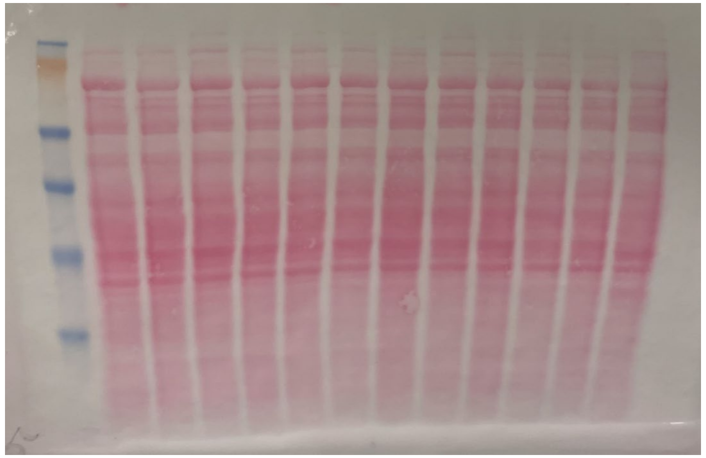

Groups:

1. WD (mice 7-8)
2. HFD + BAM15 (mice 5-6)
3. HFD + Low Semaglutide (mice 5-6)
4. HFD + Low Semaglutide + BAM15 (mice 5-6)
5. HFD + High Semaglutide (mice 5-6)
6. HFD + High Semaglutide + BAM15 (mice 5-6)

Gel 3a: FAS, AMPK, pAMPK & B-Actin

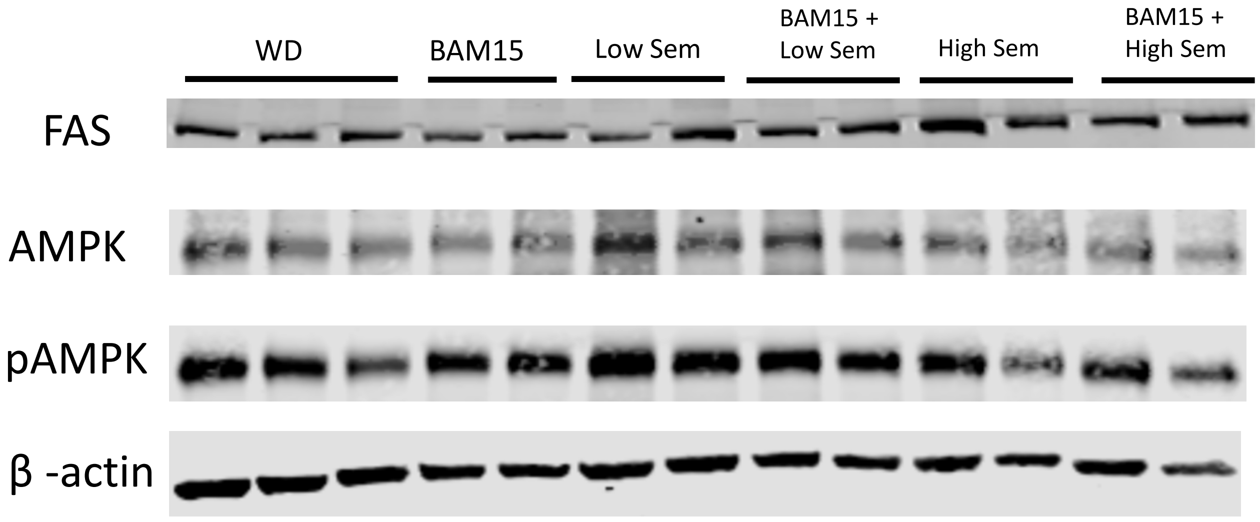

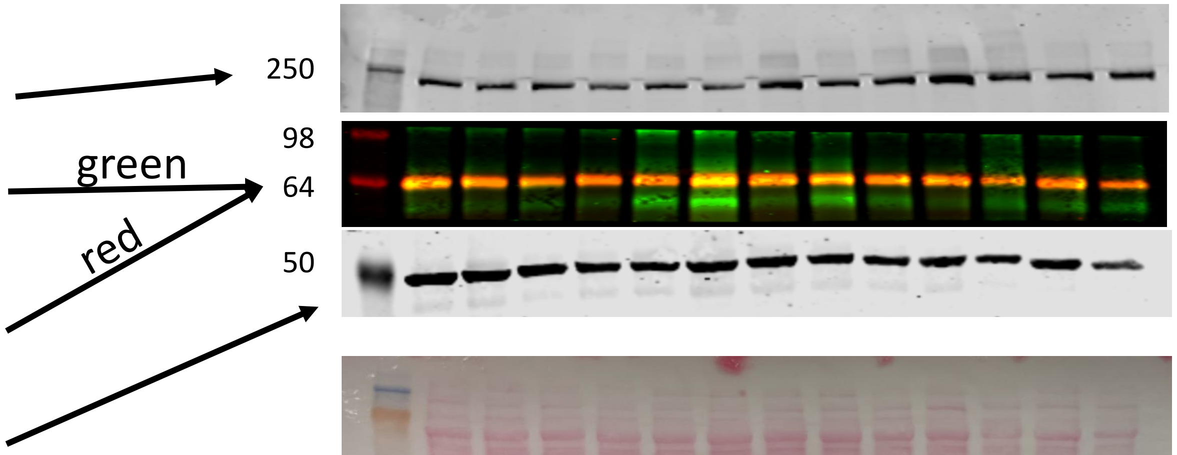

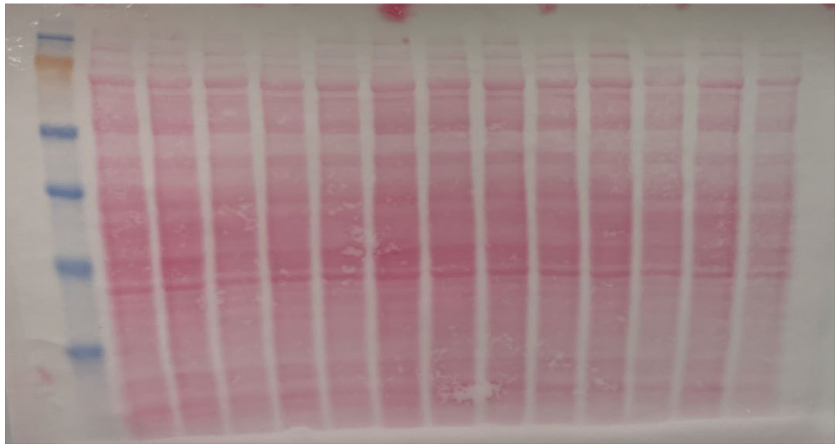

Groups:

1. WD (mice 1-3)
2. HFD + BAM15 (mice 1-2)
3. HFD + Low Semaglutide (mice 1-2)
4. HFD + Low Semaglutide + BAM15 (mice 1-2)
5. HFD + High Semaglutide (mice 1-2)
6. HFD + High Semaglutide + BAM15 (mice 1-2)

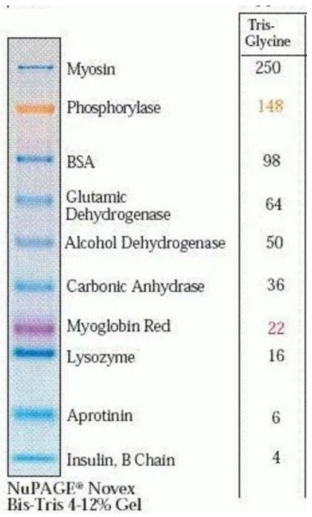

Gel 3b: FAS, AMPK, pAMPK & B-Actin

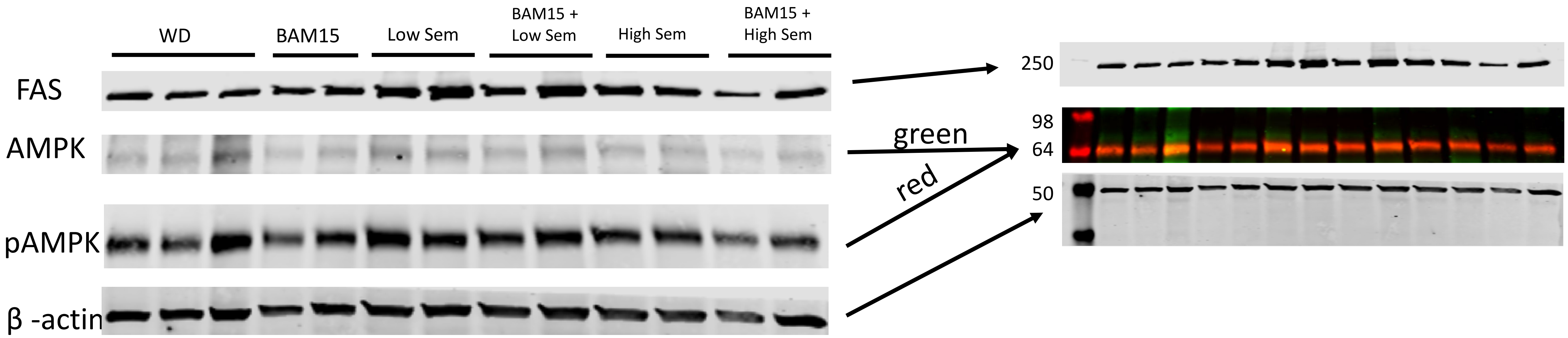

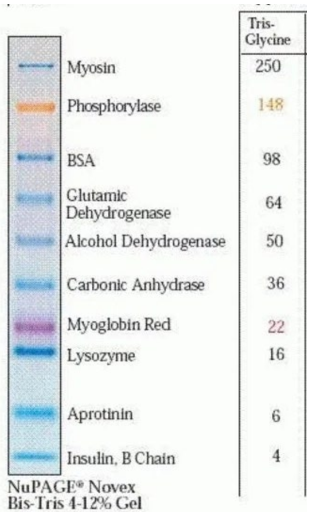

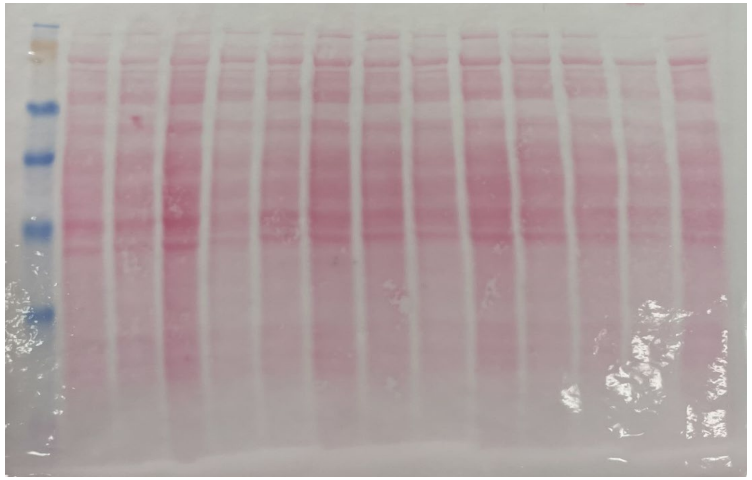

Groups:

1. WD (mice 4-6)
2. HFD + BAM15 (mice 3-4)
3. HFD + Low Semaglutide (mice 3-4)
4. HFD + Low Semaglutide + BAM15 (mice 3-4)
5. HFD + High Semaglutide (mice 3-4)
6. HFD + High Semaglutide + BAM15 (mice 3-4)

Gel 3c: FAS, AMPK, pAMPK & B-Actin

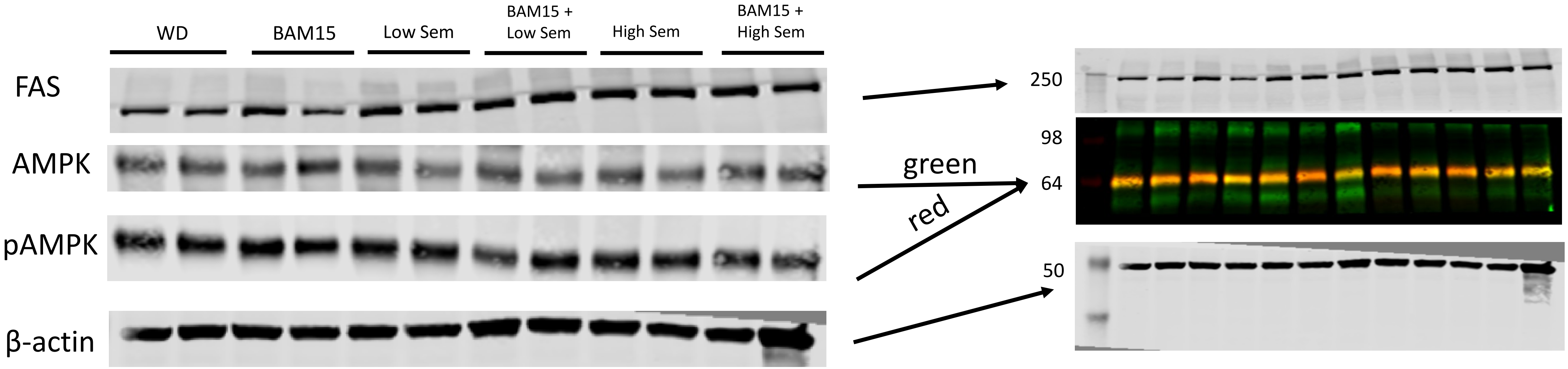

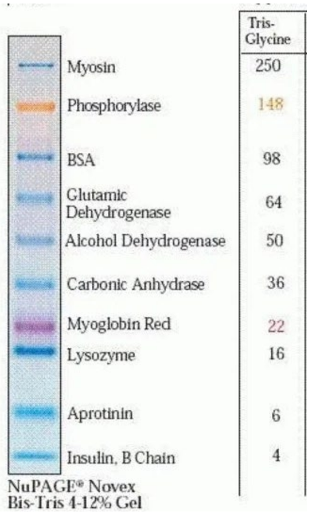

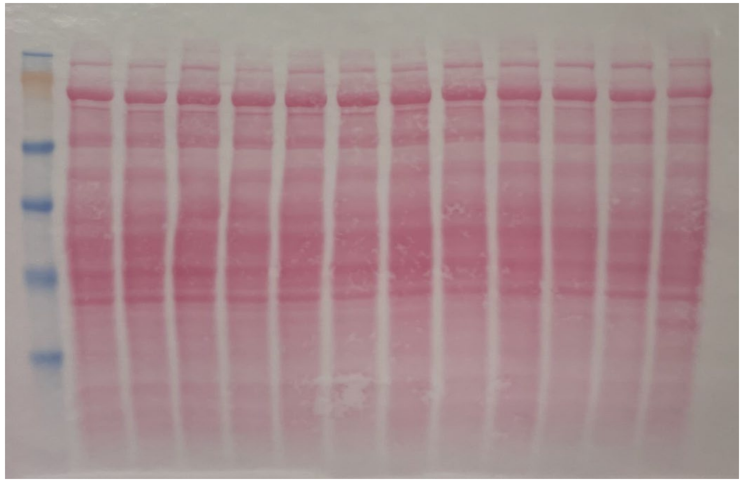

Groups:

1. WD (mice 7-8)
2. HFD + BAM15 (mice 5-6)
3. HFD + Low Semaglutide (mice 5-6)
4. HFD + Low Semaglutide + BAM15 (mice 5-6)
5. HFD + High Semaglutide (mice 5-6)
6. HFD + High Semaglutide + BAM15 (mice 5-6)
